# Selected cardiac abnormalities in *Trypanosoma cruzi* serologically positive, discordant, and negative working dogs along the Texas-Mexico border

**DOI:** 10.1186/s12917-020-02322-6

**Published:** 2020-03-30

**Authors:** Alyssa C. Meyers, Megan M. Ellis, Julia C. Purnell, Lisa D. Auckland, Marvin Meinders, Ashley B. Saunders, Sarah A. Hamer

**Affiliations:** 1 Veterinary Integrative Biosciences Department, College of Veterinary Medicine and Biomedical Sciences, Texas A&M University, MS4458, College Station, TX 77843-4458 USA; 2 College of Veterinary Medicine and Biomedical Sciences, Colorado State University, 1601 Campus Delivery, Fort Collins, CO 80523-1601 USA; 3 National Association of Federal Veterinarians, 1910 Sunderland Pl NW, Washington, D.C 20036 USA

**Keywords:** Chagas disease, Dog, Electrocardiogram, Cardiac troponin I, Zoonotic disease, Epidemiology

## Abstract

**Background:**

Chagas disease is increasingly recognized in the southern U.S., where triatomine vectors transmit *Trypanosoma cruzi* among wildlife and domestic dogs with occasional vector spillover to humans. As in humans, clinical outcome in dogs is variable, ranging from acute death to asymptomatic infections or chronic heart disease. In order to characterize cardiac manifestations of *T. cruzi* infections, we tracked a cohort of naturally-infected dogs and a matched cohort of uninfected dogs. We hypothesized that selected measures of cardiac disease (abnormal rate, abnormal rhythm, and elevated cardiac troponin I (cTnI; a biomarker of cardiac injury)) would occur more commonly in infected than uninfected dogs matched by age, breed, sex and location. In addition to the clearly positive and negative dogs, we specifically tracked dogs with discordant test results across three independent serological assays to gather clinical data that might elucidate the infection status of these animals and inform the utility of the different testing approaches.

**Results:**

We placed an ambulatory ECG monitor (Holter) on 48 government working dogs and analyzed 39 successful recordings that met length and quality criteria from 17 *T. cruzi*-infected, 18 uninfected dogs and 4 dogs with discordant results. Overall, 76.5% of positive, 100.0% of discordant, and 11.1% of negative dogs showed > 1 ECG abnormality (*p* < 0.0001), and positive and discordant dogs had a higher mean number of different types of ECG abnormalities than negative dogs (*p* < 0.001–0.014). The most common cardiac abnormalities included supraventricular and ventricular arrhythmias and atrioventricular block. Positive dogs had higher serum concentrations of cTnI than both negative dogs (*p* = 0.044) and discordant dogs (*p* = 0.06). Based on dog handler reports, nearly all (4/5; 80%) dogs with reported performance decline or fatigue were *T. cruzi*-infected dogs.

****Conclusions**:**

Further understanding cardiac manifestations in dogs naturally infected with *T. cruzi* is critical for prognostication, establishing a baseline for drug and vaccine studies, and better understanding of zoonotic risk.

## Background

Despite a century of research since the discovery of *Trypanosoma cruzi* as the etiologic agent of Chagas disease, the associations between parasite infection and disease outcome are incompletely understood, complicating the diagnosis and prognosis of Chagas disease. In humans, a subset of infected individuals develop Chagas cardiomyopathy, which can cause sudden death, arrhythmias, heart failure or ventricular aneurysms [1, 2]. A similar range of clinical outcomes is reported in dogs with no current morbidity or mortality estimates. Four mechanisms are attributed to the development of Chagas cardiomyopathy in humans: cardiac dysautonomia, microvascular disturbances, parasite induced myocardial damage, and immune-mediated myocardial injury [1], with these mechanisms relevant to dogs and other hosts as well.

In South and Central America, dogs are important sentinels and a reservoir for *T. cruzi* transmission [3–7] and are good models for understanding the pathogenesis of *T. cruzi* infection in humans. Currently, dogs and non-human primates are the only other known species that develop acute, indeterminate and chronic stages of infection similar to humans [8–11]. Chagas disease can take years to manifest after initial infection, making the use of animal models challenging and in some cases, prohibitively expensive. However, naturally-infected dogs are widespread in the southern U.S., particularly in the state of Texas [12–17], and the study of these dogs can be informative for understanding disease progression and prognostic indicators. Dogs have also served as experimental models for benznidazole treatment during the acute and chronic stage of infection [18, 19], however, a better understanding of disease progression is necessary for interpretation of drug effectiveness.

*T. cruzi* is transmitted through the introduction of infected triatomine feces through the bite site or mucous membrane, or orally through ingestion of the triatomines or their feces. The most commonly encountered triatomine in south-central Texas is *Triatoma gerstaeckeri* [20–22]. *T. gerstaeckeri* is frequently found in and around dog kennels and other peridomestic structures and has shown high rates of infection with *T. cruzi* [14, 20, 22, 23]. After an animal is exposed the trypomastigote invades the mammalian host cells [24], where *T. cruzi* has a tissue tropism for cardiomyocytes and skeletal muscle [25]. Invasion of cardiomyocytes causes cellular injury, causing release of cytokines which can have proarrhythmic effects. Persistent parasite infection of the heart causes continued stimulation of the immune response, which can cause cell death leading to replacement fibrosis and secondary hypertrophy [1]. In dogs, as in humans, electrocardiogram (ECG) abnormalities detected during *T. cruzi* infection can vary widely, and reported abnormalities in experimentally and naturally infected dogs include changes to the ECG complex morphology (i.e. T wave abnormalities, small complexes), axis shifts, conduction disturbances (i.e. atrioventricular block, bundle branch block) and arrhythmias (supraventricular and ventricular) [26, 27]. During the subacute stage clinical signs may not be evident, and ECG recordings can be normal or have minimal abnormalities [1]. Cardiac troponin I (cTnI), a biomarker of cardiac injury, can assist in the diagnosis of acute myocardial injury, and elevated cTnI has been associated with Chagas myocarditis in dogs [28]. There are currently no vaccinations or approved anti-parasitic treatments for *T. cruzi* infections in dogs in the U.S., consequently, disease prevention is focused on limiting canine contact with vectors.

The goal of this study is to identify and describe selected cardiac abnormalities associated with dogs naturally infected with *T. cruzi* as a model population for further defining parasite-host interactions. Since the cardiac abnormalities associated with Chagas disease in humans and animals are generally not specific and may also arise from other conditions, the specific comparison of selected cardiac measures between an infected and uninfected population is a useful approach for identifying abnormalities that are associated with infection status. Understanding the cardiac manifestations of natural *T. cruzi* infections in dogs is critical for prognostication and to establish a baseline for interpreting data from drug and vaccine studies.

## Results

### Study population

In 2017, 48 dogs had Holter monitors placed. Seven monitors were non-diagnostic due to dogs either chewing or removing the electrodes. Of the 41 successful recordings, 17 were from positives dogs, 6 were from discordant dogs and 18 were from negative dogs. Two discordant dogs had read times less than 19 h and were excluded from the ECG analysis. After additional testing was performed in 2017 dogs were categorized into positive, discordant and negative (as described in the methods). Due to changes in serostatus and failed recordings, the matching criteria resulted in 14 of the negative dogs individually matched to 14 positive or discordant dogs based on age, breed, sex, and location. There were an additional 6 positive dogs, 2 discordant dogs, and 3 negatives dogs enrolled in the study that lost their matches but were included in analysis. Detailed antibody testing history for each individual dog is reported in the Supplemental Table.

### Overall health

Of all 41 handlers questioned about their dog’s health, 5 (12.2%) reported that they had seen a performance decline in their dog over the past 2 years. The 5 dogs reported to have a performance decline included 4 that were *T. cruzi* positive and 1 that was negative for which the handler attributed the performance decline to the dog’s age (10 years old). Positive dogs experiencing performance decline ranged from 4.78–7.39 years. Handlers of 5 positive dogs reported fatigue (3 of these handlers had also reported performance decline); the age of dogs with fatigue ranged from 4.78–8.30 year. No handlers of discordant or negative dogs reported fatigue. Other major health concerns reported by owners included: one dog was receiving allergy shots, one dog had had a splenic torsion, one dog had chronic ear infections, one had had coccidiosis. Blood samples from all dogs were tested on the SNAP 4Dx Plus assay (Supplemental Table 1). Three dogs (7.3%) were positive for *Dirofilaria immitis* antigen, including one positive, discordant, and negative for *T. cruzi* and none of these dogs had cardiac abnormalities. One *T. cruzi*-positive dog tested positive for antibodies to *Ehrlichia* sp., and this dog had first-degree atrioventricular (AV) block and 5 VPCs per 24 h (#53).

### ECG findings

The 39 Holter analyzed recording times ranged from 19:04–48:00 h, with a median read time of 47:26. Twenty-six of the recordings were of good quality and the remaining 13 were a good quality with some baseline artifact but were still able to be interpreted. Overall, 19/39 (48.7%) dogs were characterized as having at least one ECG abnormality. When dogs were dichotomized as having 1 or more ECG abnormality vs. no abnormality, there was a significant difference between presence of abnormality and *T. cruzi* infection (*p* < 0.0001), with abnormalities detected in 76.5% (13/17) of positive dogs, 100.0% (4/4) of discordant dogs, and 11.1% (2/18) of negative dogs. Ventricular arrythmias were the most common ECG abnormality (when defined as ≥ 4.2 VPCs/24 h and a Lown score of ≥ 2), and were present in 47.1% (8/17) of positive dogs, 50.0% (2/4) of discordant dogs and 5.6% (1/18) of negative dogs (Table 1). Additional ECG abnormalities observed included first and second-degree AV block, and supraventricular premature beats/tachycardia. Neither atrial fibrillation or bundle branch block were identified in any dog.

**Table 1:** Anatomic level (atria, AV node, or ventricle) and presence of arrhythmias and conduction abnormalities found in *T. cruzi* positive, discordant and negative government working dogs

		Atria			AV node		Ventricle		
**Infection Status**	**Dog #**	**Supraventricular premature contractions**	**Supraventricular Tachycardia**	**Sinus Arrest (pauses** **>** **4 s)**	**First-degree AV Block**	**Second-degree AV Block**	**Ventricular Tachycardia**	**VPCs/24 h**	**Modified Lown Score for ventricular arrhythmias**
Positive (*n* = 17)	*238*	X						0	0
	*369*				X			0	0
	*200*			X				0	0
	*62*			X				0	0
	*159*	X						3	1
	*53*				X			5	2
	*203*							11	4
	*105*	X	X					12	1
	*60*							38	1
	*153*	X			X			79	2
	*65*						X	691	5
	*92*					X	X	1163	5
	*64*							6594	2
Discordant (*n* = 4)	*234*	X		X				0	0
	*2*					X		0	0
	*198*							25	1
	*227*							286	2
Negative (*n* = 18)	*54*			X				0	0
	*94*							1	1
	*530*							1	1
	*16*							3	1
	*193*							4	1
	*258*				X	X		762	5

*16 dogs are not displayed on the table because they had 0 VPCs and no apparent abnormalities in the 24 h monitoring. This includes 4 positive dogs and 12 negative dogs

There was a significant difference between mean number of different ECG abnormalities among *T. cruzi* infection status (*x*^2^(2) = 15.8, *p* < 0.001), in which the mean number of abnormalities was higher in both positive compared to negative dogs (*p* < 0.001), and discordant compared to negative dogs (*p* = 0.014) and there was no difference between positive and discordant dogs (*p* = 0.63, Table 2).

**Table 2:** The number of different types of electrocardiographic abnormalities found using a 24-h ECG Holter monitor in *T. cruzi* positive, discordant and negative government working dogs

	Number of different types of ECG abnormalities				Total
	0	1	2	3	
Negative	16	1	0	1	18
Discordant	0	3	1	0	4
Positive	4	8	2	3	17
**Total**	20	12	3	4	39

**Fig. 1:**
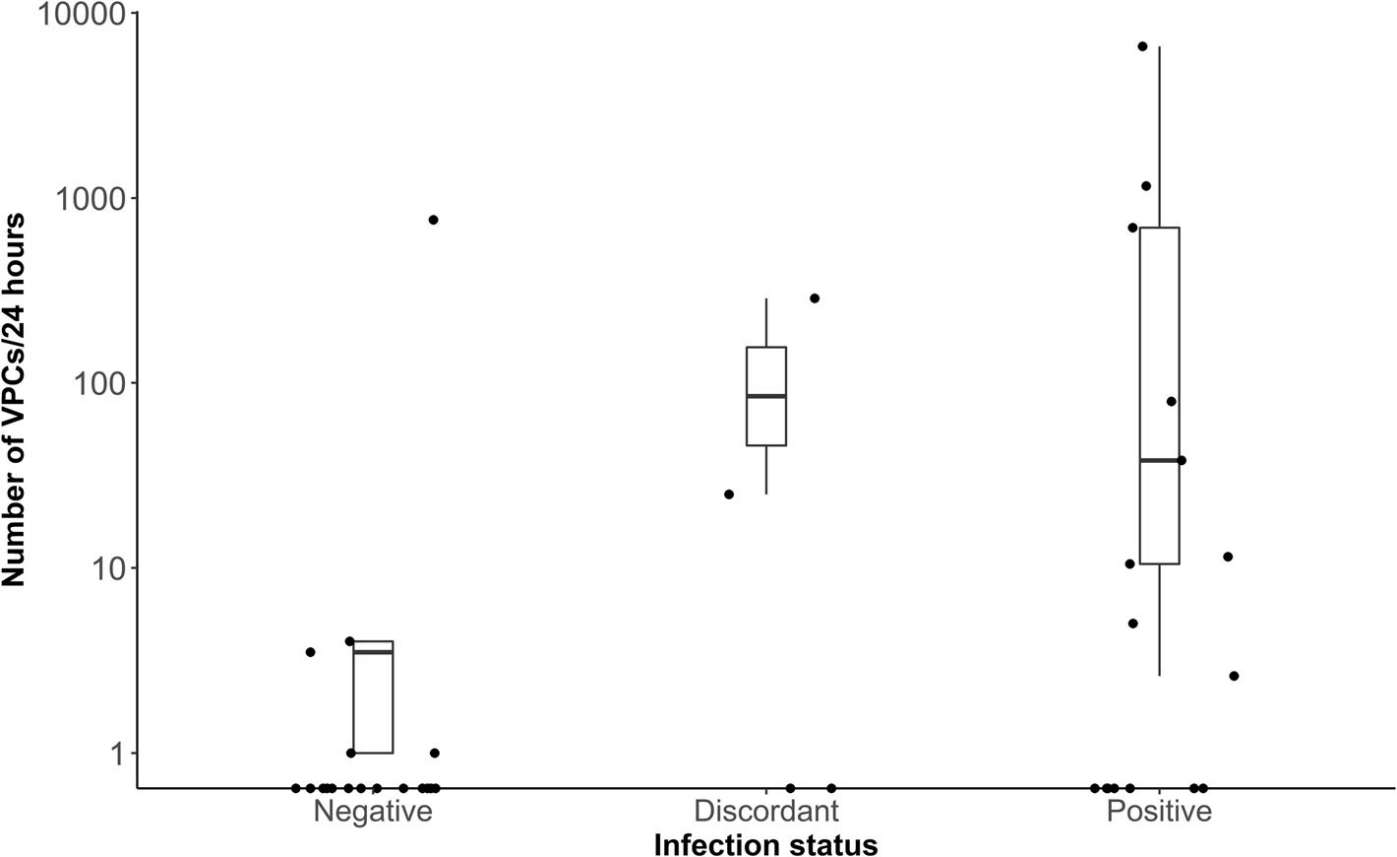
Box plots illustrating the normalized number of VPCs per 24 h in *T. cruzi* positive, discordant and negative government working dogs. Y-axis scaled to log10

The number of VPCs present in 24-h was not significantly different across *T. cruzi* status (*x*^2^ (2) = 4.58, *p* = 0.10). Number of VPCs ranged from 0 to 4 in negative dogs, with one outlier that had 762 VPCs/24 h. The discordant dogs ranged from 0 to 286, and the positive dogs ranged from 0 to 6594 VPCs/24 h (Table 1, Fig. 1).

All dogs were assigned a modified Lown score quantifying the severity of the ventricular arrhythmias present. Overall, 35.3% of *T. cruzi* positive dogs had a Lown score of 2 or more compared to 25.0% of discordant dogs and 5.5% of negative dogs (*x*^2^ (2) = 3.27, *p* = 0.20, Table 3).

**Table 3:** Modified Lown score for severity of ventricular arrhythmias found on ambulatory ECG (Holter) from *T. cruzi* positive, discordant and negative government working dogs

		Modified Lown Score*					
	Total dogs	0	1	2	3	4	5
Negative	18	13	4	0	0	0	1
Discordant	4	2	1	1	0	0	0
Positive	17	8	3	3	0	1	2
**Total dogs**	39	22	8	4	0	1	3

*0- no VPCs; 1-single uniform VPCs; 2-bigeminy, trigeminy, or multiform VPCs; 3- accelerated idioventricular rhythm; 4- VPCs in couplets or triplets; 5- “R on T” phenomenon or ventricular tachycardia

Quantitative variables from the ECG including heart rate range and pauses are displayed in Table 4. Maximum heart rate was not significantly different between positive and negative dogs (*p* = 0.11), positive and discordant dogs (*p* = 0.98) or between discordant and negative dogs (*p* = 0.32). Analysis on all other quantitative variables showed no significant difference among *T. cruzi* infection status (Table 4). Overall, there were 11.8% (2/17) positive dogs that had more than 25 pauses over 3 s during the time observed, and no negative or discordant dogs with more than 25 pauses over 3 s. Sinus arrest > 4 s was the reason for pauses in 4 dogs (#200, 62, 234 and 54); two of these dogs were positive, one was discordant and one was negative.

**Table 4:** Quantitative variables found on ECG from *T. cruzi* positive, discordant and negative government working dogs

ECG variable	Positive (***N*** = 17)	Negative (***N*** = 18)	Discordant (***N*** = 4)	ANOVA ***P***-Value
**Maximum HR (bpm)**	218.5 (131–285)	245.5 (206–308)	214.8 (173–264)	0.09
**Minimum HR (bpm)**	28.3 (18–56)	28.4 (20–45)	24.3 (22–28)	0.58
**Mean HR (bpm)**	76.4 (54–124)	76.4 (58–103)	69.8 (64–79)	0.65
**Pauses > 3 s**	14.8 (0–164)	2.7 (0–25)	3.8 (1–10)	0.40
**Longest RR Interval(s)**	3.0 (1.7–4.2)	2.9 (1.7–4.1)	3.4 (3.1–4.1)	0.40

Mean and range is reported for each variable. *HR* Heart Rate, *bpm* beats per minute

**Fig. 2:**
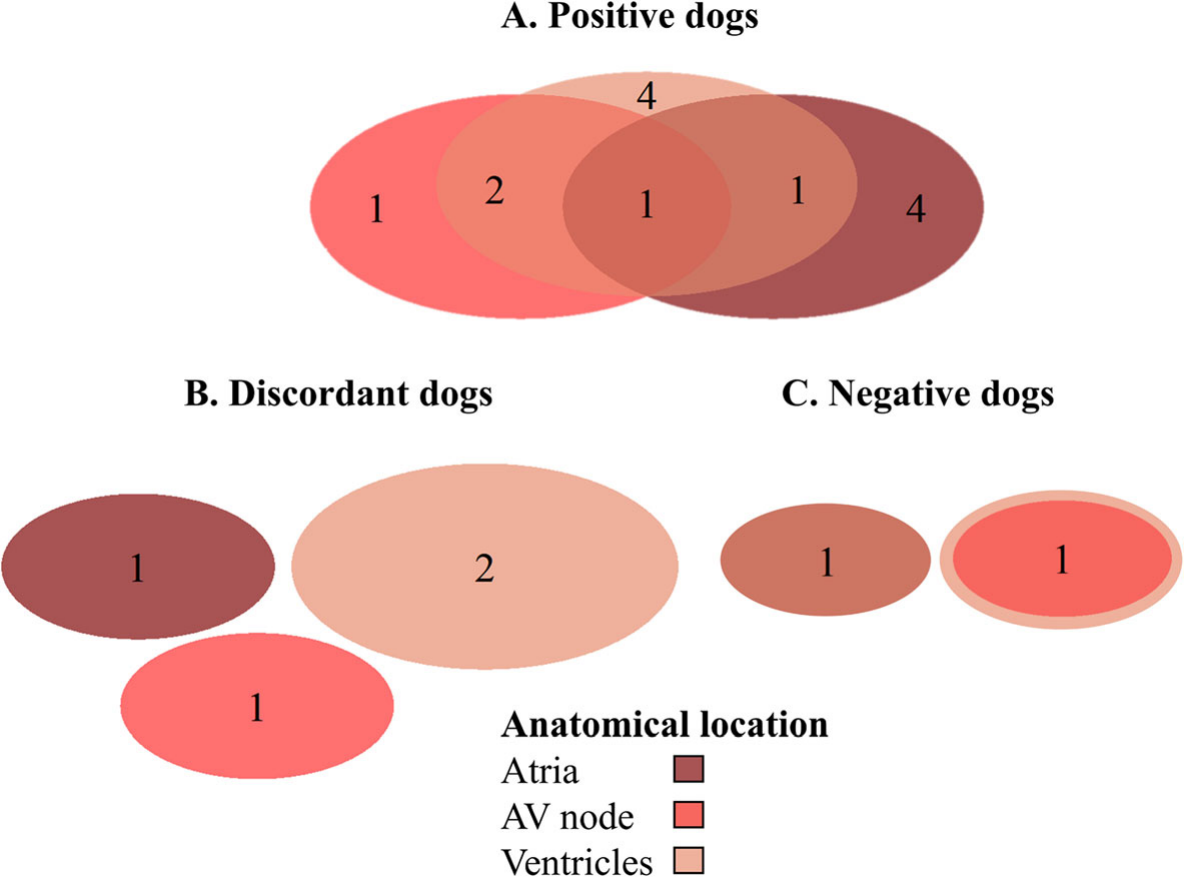
Venn diagrams demonstrating the anatomic level (atria, ventricles and/or AV node) of cardiac abnormalities in *T. cruzi* positive (**a**), discordant (**b**) and negative (**c**) government working dogs

*T. cruzi*-positive, discordant, and negative dogs showed ECG abnormalities at all three anatomic levels (atria, AV node or ventricles). Of the 39 dogs tested, some dogs demonstrated cardiac disease in one location, two locations or all three. Individual dogs with abnormalities present at multiple levels were more common in the positive dogs compared to the discordant and negative dogs (Fig. 2).

### Molecular analysis

All 41 dogs were negative for *T. cruzi* by PCR in 2015. Forty of these dogs remained negative by PCR in 2017. A single dog, an 8-year-old female Belgian Malinois from Rio Grande Valley management area, was PCR-positive in 2017 with a cycle threshold (CT) value of 31.32 on the Cruzi 1/2/3 real-time assay. The discrete typing unit present was TcI. This dog was serologically positive with an IFA titer of 320 and was positive on both Chagas Stat-Pak® and Chagas Detect™ in both 2015 and 2017.

### Cardiac biomarker

**Fig. 3:**
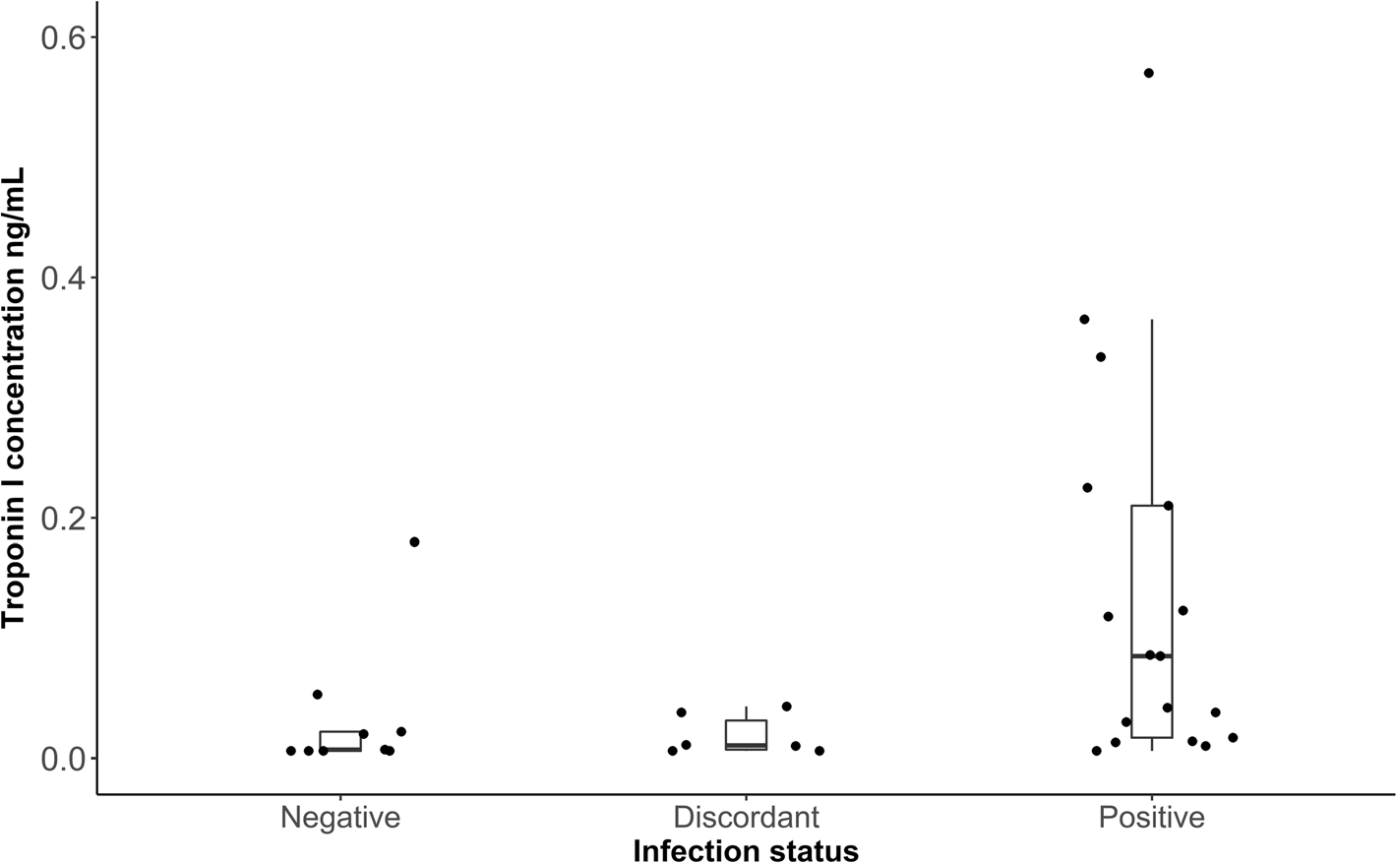
Box plots illustrating the range of cardiac troponin I concentrations in *T. cruzi* positive, discordant and negative government working dogs. Median cardiac troponin I concentration was 0.085 for positive dogs (*N* = 17), 0.007 for negative dogs (*N* = 9) and 0.011 for discordant dogs (*N* = 6)

All positive (*n* = 17) and discordant (*n* = 6) dogs, and a random subset of negative (9/18) dogs had serum submitted to measure cTnI concentrations. The cTnI concentrations ranged from < 0.006 ng/mL to 0.57 ng/mL in the positive dogs with a median of 0.085 ng/mL; while the discordant dogs ranged from < 0.006 ng/mL to 0.043 ng/mL, with a median of 0.011 and the negative dogs ranged was from < 0.006 ng/mL to 0.18 ng/mL, with a median of 0.007 ng/mL (Fig. 3). The cTnI level was below the limit of detection in 5.9% (1/17) of positive dogs, 33.3% (2/6) of discordant dogs and 22.2% (4/9) of negative dogs. There was a significant difference between *T. cruzi* infection status and the cTnI concentration (*x*^2^ (2) = 8.22, *p* = 0.016) with higher concentrations in positive than negative dogs (*p* = 0.044) and discordant dogs (*p* = 0.06), and no difference between discordant and negative dogs (*p* = 0.96).

## Discussion

The clinical manifestations for Chagas disease in dogs are variable and poorly understood, with most canine models of experimental infection focused in the acute infection period. Our previous research has shown that working dogs have widespread exposure to *T. cruzi* along the Texas-Mexico border with 7.4–18.9% seroprevalence depending on test interpretation (*n* = 528) [14]. Additionally, 45% of triatomines found in the working dog’s environments were infected with *T. cruzi,* and the majority had recently fed on canines [14]. Despite this high level of exposure and vector contact, many infected dogs continue to work regularly with no perceptible adverse health outcomes noted by handlers. In the current study, we aimed to quantify selected measures of cardiac health in relation to *T. cruzi* infection status among these high value government working dogs that may be exposed and re-exposed regularly.

Holter (24-h ambulatory ECG) monitors are a useful tool for monitoring the cardiac rate and rhythm abnormalities of *T. cruzi*-infection in hosts. We found that the presence (*p* < 0.0001) and number (*p* < 0.001) of ECG abnormalities was higher is seropositive dogs than seronegative dogs. Supraventricular premature contractions, AV block, ventricular arrhythmias (premature beats and tachycardia) were more commonly found in seropositive dogs than seronegative dogs. Two negative dogs had cardiac abnormalities, one dog had sinus arrest the other had numerous and polymorphic VPCs and first and second-degree AV block. A low level of ECG abnormalities in healthy populations of dogs has been described in previous studies, including sinus arrest, ventricular arrythmias and second-degree AV block [29–32]. Barr et al. 1992 found that experimentally infected beagles in the acute stage had AV block and in the chronic stage had multiform VPCs and ventricular tachycardia [26]. Other studies in dogs have also reported first-degree AV block and ventricular conduction disturbances [8, 33, 34]. Second-degree AV block, which we observed in only 1 *T. cruzi* positive dog, has been less commonly reported; prior studies have found second-degree AV block in 1/18 of *T. cruzi* infected dogs and third-degree AV block in 1/10 infected dogs [26, 27]. Some arrhythmias previously documented in dogs and humans with Chagas disease, such as bundle branch block, atrial fibrillation and third-degree AV block, were not found in this study [8, 26, 27, 33, 35]. It is possible that an expanded sample size or tracking of disease progression over time may reveal these abnormalities not described here. It is important to determine the prevalence and type of cardiac abnormalities in *T. cruzi* infected dogs to better predict clinical outcomes so future treatments can be evaluated.

Overall 76.5% (13/17) of seropositive dogs had ECG abnormalities during the study period. This is in-line with previous study findings where 90–100% of *T. cruzi* infected dogs have cardiac abnormalities [27, 36]. This percentage is higher than what has been reported for humans in which approximately 20–40% of infected people develop cardiac abnormalities resulting in heart failure, arrhythmias, heart block, thromboembolism, stroke and sudden death [37–39]. This difference in percentage of dogs that development cardiac abnormalities could be due to host immune response, physiology, exposure route or dose of parasite upon exposure. Dogs have an affinity to eat bugs, and oral transmission in dogs from the consumption of infected kissing bugs has been suggested as a likely route [15, 40–42], which could expose them to a higher density of parasite. Previous studies have shown that pathogenicity in mice and dogs is relative to the quantity or parasite inoculated and to the route of inoculation [43–45]. Specifically, metacyclic trypomastigotes have been shown to be more efficient by oral than cutaneous routes in mice [46]. In humans, oral infection with *T. cruzi* is associated with a high mortality rate (8–35% compared to < 5–10% when transmitted by the vector) [47] and an attack rate of up to 100% [48]. Research exploring if oral infection in dogs mimics more severe clinical outcomes seen in orally-infected humans compared to those infected via the stercorarian (vector-fecal) route would be useful in learning the similarities and differences between canine and human disease manifestation.

There is no gold standard diagnostic test for Chagas disease in humans or animals and so samples are commonly subjected to multiple independent tests and conflicting test results are commonly reported [12–14, 49–51]. We found that ‘discordant’ dogs – dogs that were positive on a single but not multiple independent tests- were somewhat intermediary with respect to their clinical status. Notably, we found that all four discordant dogs in our study showed ECG abnormalities, with abnormalities occurring in all anatomic levels of the heart. This is evidence that the result of a single test (in some cases a single immunochromatographic test used in an off-label manner) may be useful in signaling the potential for clinical disease and should not immediately be dismissed. Further research on a larger population of dogs, or over a longer time period, is warranted to better understand clinical outcomes in a population of discordant dogs.

We found that ventricular arrythmias were the most common cardiac abnormality seen in the working dogs. The number of VPCs/24 h and complexity of VPCs is frequently used in veterinary medicine to evaluate clinically significant ventricular arrhythmias [52–55]. Determining a threshold value of an acceptable number of VPCs above which would signal an abnormality is not straightforward. A low number of VPCs has been suggested to be normal in dogs as it is in humans [31, 32, 56] however, repetitive, numerous or polymorphic VPCs could be suggestive of cardiac disease or increased risk of sudden death. Furthermore, a study evaluating large-breed healthy dogs by ambulatory ECG for > 20 h found that only 8% (*n* = 50) had a Lown score of 2 or more and concluded this could be suggestive of cardiac or systemic disease [57]. By characterizing the data from seronegative dogs, we established a within-study threshold and categorized as abnormal dogs with ≥ 4.2 VPCs/24 h and a Lown score of ≥ 2; using these criteria, nearly half (47.1%) of the seropositive dogs had abnormal ventricular arrhythmias. Similarly, in humans with Chagas disease VPCs are one of the most common ECG abnormalities [58]. Barr et al. found that 100% of dogs inoculated with *T. cruzi* developed ventricular arrhythmias, which were polymorphic and developed into ventricular tachycardia [26]. In seronegative dogs, 27.8% (5/18) of dogs had VPCs, but only 5.6% (1/18) were above the threshold of what was established as normal for this population. Our findings of VPCs in a healthy population of dogs is in-line with previous findings of the presence of VPCs in 21.5% (*n* = 228) of clinically normal beagles, 12.5% (*n* = 16) and in a healthy population of pet dogs, and 32.0% in 50 healthy dogs [31, 32, 57].

Heart rate is related to the intensity of cardiovascular workload and can be useful for monitoring animals [59, 60]. We found no significant associations between the quantitative variables from the ECG and infection status in dogs. A previous study found that heart rate (beats per minute) in *T. cruzi* experimentally infected dogs was significantly higher than control dogs during acute infection, but no difference was found during the indeterminate phase [33]. Chronic Chagas cardiomyopathy in humans can cause a decreased response to exercise and stress due to failure of cardiac compensation [61, 62]. However, benefits of exercise training in chronic heart failure patients has been shown, and new research suggests that regular exercise could assist with cardiac function in chronic Chagas disease in humans [63, 64]. A randomized trial comparing humans with chronic Chagas disease who underwent a 12-week exercise training program compared to those who did not, found that those who completed exercise training had improvements in oxygen uptake, exercise time, walking distance and heart rate peak [63]. Since the DHS working dogs are regularly exercising, it is possible that routine exercise could benefit cardiac function similar to what is demonstrated in humans, but more research is needed to explore this.

When we examined anatomic level of ECG abnormality (atria, AV node or ventricles) we found that in all populations of dogs (positive, discordant, negative) abnormalities were present at each level, however, positive dogs were more likely to have abnormalities detected at multiple levels of the conduction system within the heart. The slow progressing myocarditis that results from infection can cause decreased contractile function and dilatation of all four chambers [48]. Histological evidence in humans and dogs shows that there is destruction of myocardial cells throughout the heart and scarring of the conduction system [28, 34, 65]. In *T. cruzi* infection focal inflammation causes destruction of cardiac fibers and subsequent fibrosis in nonregenerating cells which can result in myocardial dysfunction, heart failure and arrhythmias [35, 66].

Cardiac troponin I is an indicator of myocardial damage, and previous studies have shown that dogs and humans with Chagas cardiomyopathy can have elevated cTnI concentrations [28, 67]. We found that positive dogs had higher cTnI concentrations than discordant dogs (*p* = 0.06) and negative dogs (*p* = 0.044). cTnI can be elevated for multiple reasons associated with cardiac disease, so alone it is not a dependable test for Chagas disease screening. However, used in conjunction with serology and ECG, it provides insight into the presence of cardiac damage and could assist in diagnosis of Chagas disease [28].

This study is limited in that ECGs were only taken from one time point. To better understand the clinical outcomes of *T. cruzi* infection additional ECGs would be beneficial to assess progression and variability at future time points. Since echocardiograms were not performed on these dogs, it is possible that this study underrepresents structural and functional cardiac abnormalities. These dogs are working at checkpoints along the U.S. border and are likely exposed to other agents or environments that could impact cardiac health. To account for this potential variation, we attempted individual-level matching of positive and negative dogs in our study design and achieved age/breed/sex/location matching of 14 positive and discordant dogs to 14 negative dogs, however a match of all study dogs was not possible due to equipment failure. All DHS dogs were reported to receive monthly flea, tick and heartworm preventative and dogs had been tested annually or bi-annually for common parasites including *D. immitis, Borrelia burgdorferi, Ehrlichia* spp., and *Anaplasma* spp. To rule out cardiac abnormalities caused by these pathogens, we tested dogs using the IDEXX 4DX. Three dogs were positive for *D. immitis* but did not have ECG abnormalities and based on prior negative testing were thought to have been infected within the past year. One *T. cruzi* positive dog had antibodies to *Ehrlichia spp.,* undetectable cTnI concentration (< 0.006 ng/mL), and ECG abnormalities that included first-degree AV block and VPCs (Lown score 2). A study of dogs naturally infected with *E. canis* found that 3.33% (*n* = 150) of infected dogs had first-degree AV block, and 2.7% had VPCs [68]. This study also found that dogs infected with *E. canis* had higher concentrations of cTnI than uninfected dogs [68]. While we cannot rule out that this dog’s cardiac abnormalities were due to *E. canis*, the dog had a history of being infected with *T. cruzi* since 2013 (4 years), whereas the infection with *E. canis* was likely in the past year since dogs are checked annually for *E. canis*. Co-infections with multiple pathogens can cause complex interactions within a host and could have modified immunopathological outcomes. Limited research has been done on co-infection in dogs with *T. cruzi* and further research is warranted. This study is further limited by the ECG tracings being read by automatic analysis. Although all tracings were reviewed and abnormalities confirmed by a board-certified veterinary cardiologist, it is possible abnormalities were missed in the automatic analysis. Finally, supraventricular premature beats were reported as being present or absent and the number/24 h was not reported. A scoring system for supraventricular arrhythmias is not available and use of the modified Lown score was not readily applicable.

The DHS working dogs play a critical role in border security, and the cardiac manifestations of Chagas disease are likely associated with a yet-unquantified impact on their ability to work with economic and security consequences. In a broader application, understanding the cardiac manifestations of natural *T. cruzi* infections in dogs is critical for prognostication and to establish a baseline for interpreting data from intervention and treatment studies. We demonstrated that *T. cruzi* infected dogs that appeared healthy to their handlers had significantly higher cTnI and multiple ECG abnormalities compared to negative dogs. Our research highlights the need for routine testing by ECG and serum concentrations of cTnI in dogs that are antibody positive for *T. cruzi*. Management of ECG abnormalities could improve survival. As there are many causes of myocarditis in dogs, it is essential to build veterinary awareness and define an index of suspicion of when and where to test dogs for *T. cruzi*. Further, owner education on vector identification and environmental risk factors could also reduce exposure and increase timely diagnosis in dogs. As a model of naturally and locally-infected hosts across the landscape, understanding the epidemiology and clinical outcomes of *T. cruzi* infection in these dogs can advance not only veterinary but also human medicine.

## Conclusions

When comparing ambulatory ECG monitor (Holter) recordings from 17 *T. cruzi*-infected, 18 uninfected dogs and 4 dogs with discordant results we found the presence (*p* < 0.0001) and number (*p* < 0.001) of ECG abnormalities was higher in infected verses uninfected dogs. Furthermore, we found that infected dogs had higher serum concentrations of cTnI than both negative dogs (*p*= 0.044) and discordant dogs (*p* = 0.06). These combined results suggest that *T. cruzi* infection can cause ECG abnormalities and an elevation in cTnI.We also found the most common ECG abnormalities in positive dogs to included supraventricular premature contractions, AV block, and ventricular arrhythmias (premature beats and tachycardia). Determining the type of cardiac abnormalities in *T. cruzi* infected dogs and the frequency allow for better prediction of clinical outcomes and evaluation of possible treatments.

## Methods

### Study population- working dogs along the Texas-Mexico border

The Department of Homeland Security (DHS) of the U.S. government utilizes highly trained dogs for border security and detection purposes. DHS working dogs are predominantly bred in Europe, with the exception of Labrador Retrievers which are bred domestically. DHS working dogs receive 6 months training at a working dog training facility either in El Paso, Texas, or Front Royal, Virginia. Once training is complete, dogs are assigned to work at specific locations across the U.S. and have limited travel. Dogs included in this study worked either immediately adjacent to the geopolitical Texas-Mexico border (ports of entry) or north of the border (checkpoints). All dogs receive vaccines and deworming annually, are on tick and flea preventative and receive bi-annual health exams. A total of 48 dogs were enrolled, but only 41 had interpretable data for analysis. Polymerase chain reaction (PCR) results and serology known at the time of selection are summarized in supplemental Table 1. The majority of 41 dogs were male (*n* = 26). Age ranged from 3.3–11.2 years with the average age of 6.6 years and median of 7.0. Breeds sampled included Belgian Malinois (*n* = 22), German shepherd (*n* = 11), Dutch shepherd (*n* = 3), Labrador retriever (*n* = 2), Sable Shepherd (n = 2) and Groenendael (n = 1). Dogs worked in 3 management locations in Texas: Del Rio (*n* = 14), Laredo (*n* = 9) and the Rio Grande Valley (*n* = 23). When not performing working duties, most of the dogs (*n* = 32) were housed at home with their handlers, but 9 were group kenneled.

Dogs were selected from a population of DHS working dogs that were initially tested for *T. cruzi* DNA and antibodies by our research team in November of 2015 [14], and we performed follow-up testing in 2017. In addition to PCR, we use up to three independent serology tests on each dog per year, and require a positive reaction with at least two serology tests to code a dog as positive in a given year. Two exceptions were made with two dogs which had a history of being positive on indirect fluorescent antibody (IFA) prior to the start of our study and then had positive reactions on at least one test in both 2015 and 2017 (methods below). We classified dogs into three categories for analysis: ‘positive’ (positive test result on two or more assays in 2015 or IFA testing prior to 2015 and at least one test in both 2015 and 2017); ‘discordant’ (either (i) positive on only a single test each year (ii) or had a history of being IFA-positive prior to 2015 testing but negative on all tests in 2015 and 2017); and ‘negative’ (negative on all tests in 2015 and 2017 and no history of positivity prior to our study). Dogs were initially enrolled and matched (positive and discordant dogs to negative dogs) based on age (+/− 1 year), sex, breed and location of sector (Del Rio, Laredo and Rio Grande Valley) based on their 2015 testing. In two cases, positive or discordant German Shepherds could not be matched by breed, and were instead matched to a negative Sable Shepherd and Dutch Shepherd. In addition to negative serology, all negative dogs were required to be PCR negative as a step to reduce the chance that acutely infected dogs (i.e., prior to development of detectable antibodies) were classified as negative.

### ECG monitoring

To characterize cardiac arrhythmias and conduction abnormalities, we applied a 5-lead continuous read ambulatory ECG ‘Holter’ monitor (LabCorp, Burlington, NC). This model has previously been used in both canine and human medicine, and records heart rate, pauses, R-R variability (total duration of ventricular depolarization) and rhythm and conduction abnormalities. On each dog, an area was shaved and cleaned with alcohol for electrode placement. Electrodes were placed according to manufacturer instructions on the lateral chest walls of the dog. The electrodes and battery were secured using 3-in. Ultra-Light™ elastic adhesive tape (Covidien, Dublin, Republic of Ireland), over which a working vest was placed. Dogs conducted their normal working routine to provide an accurate representation of daily cardiac stress. Monitors were removed after 24–48 h. Tracings obtained from the ECG were recorded at a speed of 25 mm/s using a lead configuration of V1, V2 and V5, then transferred to LabCorp for automatic analysis. During ECG application, each canine handler was asked if he or she had observed a change in performance in their dog, or fatigue, or if the dog had any other major health concerns over the past 2 years. All responses were recorded.

Full disclosure tracings were reviewed by a board-certified veterinary cardiologist (ABS) who was blinded to the dog’s infection status to characterize the occurrence and severity of arrhythmias and conduction abnormalities. Recordings made unreadable by excessive background noise or artifact, or with read times less than 19 h were excluded from further analysis. The following were recorded: analyzed duration, heart rate (max, min, mean), number of pauses > 3000 ms, longest RR interval, and sinus arrest > 4 s. The presence of ventricular premature contractions (VPCs) was recorded and the number was normalized to 24 h. Ventricular arrhythmias were graded based on a modified Lown score using the following criteria: 0- no VPCs; 1-single uniform VPCs; 2-bigeminy, trigeminy, or multiform VPCs; 3- accelerated idioventricular rhythm; 4- VPCs in couplets or triplets; 5- “R on T” phenomenon or ventricular tachycardia [52, 69]. Also recorded were presence of supraventricular arrhythmias and presence and degree of atrioventricular block. Supraventricular and ventricular tachycardia were defined as > 3 abnormal complexes in a row at a heart rate of > 100 bpm if greater than 4 beats. The anatomic level of the ECG abnormality was recorded as being present or absent at the atria, atrioventricular (AV) node, or ventricle. The following ECG abnormalities were assigned as being at the anatomical level of atrial (supraventricular premature beats, supraventricular tachycardia including atrial fibrillation, sinus arrest > 4 s), AV node (atrioventricular block – any degree), and/or ventricle (ventricular premature beats, ventricular tachycardia, bundle branch block).

### Serologic and molecular testing

At the time the Holter monitor was applied, a minimum of 6 ml of blood was collected and aliquoted into serum and ethylenediaminetetraacetic acid (EDTA) tubes, spun, separated, and stored at -20 °C until analysis. All dog serum was tested for anti-*T. cruzi* antibodies by two rapid immunochromatographic assays, Chagas Stat-Pak® (ChemBio, NY), and Chagas Detect™ Plus Rapid Test (InBios, International, Inc., Seattle, WA) using previously described methods [14]. All dogs that gave a positive result on one or both rapid tests were also tested on indirect fluorescent antibody (IFA) test at the Texas Veterinary Medical Diagnostic Laboratory (TVMDL), College Station, TX. Titer values of 1:20 or higher were considered positive as per TVMDL standard protocol.

All dogs were tested for amplification of parasite DNA from buffy coat of blood by real-time PCR as previously described [70, 71]; in the case of a positive rt-PCR reaction, additional rt-PCR was carried out to assign the parasite discrete typing unit [72]. All deviations from protocols are previously described [14, 73].

To rule out cardiac abnormalities caused by other vector-borne diseases, we used a commercially available rapid format enzyme-linked immunosorbent assay (ELISA), the SNAP 4Dx Plus (IDEXX, Westbrook, ME), for detection of *Dirofilaria immitis* (heartworm) antigen and antibodies to *Ehrlichia canis*, *E. ewingii*, *Borrelia burgdorferi*, *Anaplasma. phagocytophilum*, and *A. platys*.

### Cardiac biomarker

Frozen serum samples were slowly thawed at room temperature immediately prior to analysis. cTnI analysis was performed using an ADVIA Centaur TnI-Ultra immunoassay at the Gastrointestinal Laboratory at Texas A&M University on 250 uL of serum. The reported range for cTnI detection by the manufacturer is 0.006 to 50.0 ng/mL.

### Statistical methods

To evaluate the relationship between dog *T. cruzi* infection status (positive, discordant, or negative) and clinical outcomes, data were imported into R 3.4.2 (2017-09-28) software for analysis. Bivariable analysis using Fisher’s exact was used to determine if *T. cruzi* infection was associated with the presence of a cardiac abnormality. Since there is no pre-defined threshold number of VPCs/24 h that is accepted as normal in healthy dogs, we used the seronegative dog population to establish a within-study threshold value of 4.2 VPCs/24 h; this threshold value was calculated as three standard deviations above the mean from negative dogs (μ = 0.56, *σ* =1.2). One negative dog (#258) had 762 VPCs/24 h and was considered an outlier and was removed from the calculation for a threshold. VPCs were included as a cardiac abnormality in analysis when there was > 4.2 VPCs/24 h and/or the modified Lown score was 2 or more. Quantitative variables from the ECG were analyzed using one-way analysis of variance (ANOVA) followed by a Tukey’s test. For variables that were not normally distributed, statistical significance was determined by the non-parametric Kruskal Wallace ranked sum followed by Dunn’s test with a Holm adjustment of *p*-values. This includes the evaluation of infection and the number of cardiac abnormalities, modified Lown score, number of VPCs/24 h and cTnI concentration. Significance was defined as *P* ≤ 0.05.

## Supplementary information

**Additional file 1: Table S1.** Demographics and test results for government working dogs. Serology included testing for anti-*T. cruzi* antibodies by two rapid immunochromatographic assays, Chagas Stat-Pak® (ChemBio, NY), and Chagas Detect™ Plus Rapid Test (InBios, International, Inc., Seattle, WA), indirect fluorescent antibody (IFA) testing at Texas Veterinary Medical Diagnostic Laboratory (College Station, TX) and a commercially available ELISA, the SNAP 4Dx Plus, which allows for simultaneous detection of canine antibodies to *E. canis*, *E. ewingii*, *B. burgdorferi*, *A. phagocytophilum*, and *A. platys*, and to *D. immitis* antigen. Dogs were also tested for amplification of *T, cruzi* DNA by real time PCR.

## Data Availability

The datasets generated and/or analyzed during the current study are not publicly available because they contain law-enforcement sensitive data but are available from the corresponding author on reasonable request.

## Abbreviations

ANOVA: Analysis of variance
AV: Atrioventricular
*cTnI*: *Cardiac* troponin I
DHS: Department of Homeland Security
ECG: Electrocardiogram
EDTA: Ethylenediaminetetraacetic acid
ELISA: Enzyme-linked immunosorbent assay
*IFA*: Indirect fluorescent antibody
*PCR*: Polymerase chain reaction
TVMDL: Texas A&M Veterinary Medical Diagnostic Laboratory
*VPC*: Ventricular premature contractions
