# Supplementary material for: Selected cardiac abnormalities in Trypanosoma cruzi serologically positive, discordant, and negative working dogs along the Texas-Mexico border
Source: BMC Vet Res. 2020 Mar 30;16:101. doi: 10.1186/s12917-020-02322-6 (PMC7106864; doi:10.1186/s12917-020-02322-6)
Supplement: Supplementary file 1 — Additional file 1: Table S1. Demographics and test results for government working dogs. Serology included testing for anti-T. cruzi antibodies by two rapid immunochromatographic assays, Chagas Stat-Pak® (ChemBio, NY), and Chagas Detect™ Plus Rapid Test (InBios, International, Inc., Seattle, WA), indirect fluorescent antibody (IFA) testing at Texas Veterinary Medical Diagnostic Laboratory (College Station, TX) and a commercially available ELISA, the SNAP 4Dx Plus, which allows for simultaneous detection of canine antibodies to E. canis, E. ewingii, B. burgdorferi, A. phagocytophilum, and A. platys, and to D. immitis antigen. Dogs were also tested for amplification of T, cruzi DNA by real time PCR. [file 12917_2020_2322_MOESM1_ESM.pdf]

Demographics							2015 testing				2017 testing					4Dx testing			
Infection Status	Dog #	Sex	Breed	Age	Sector	Sleeping Location	StatPak	IFA	InBios	rt-PCR	StatPak	IFA	InBios	rt-PCR	Pre-study history of IFA	*Dirofilaria immitis*	*Borrelia burgdorferi*	Ehrlichia spp.	Anaplasma spp.
Discordant	211	M	Belgian Malinois	6.2	Del Rio	Kennel	-	<20	+	-	-	<20	+	-		-	-	-	-
	234	F	Belgian Malinois	10.5	Del Rio	Home	-	<20	+	-	-	<20	+	-		-	-	-	-
	100	M	Belgian Malinois	5.7	RGV	Home	-	<20	-	-	-	<20	-	-	2012-1:20	+	-	-	-
	2	M	German Shepherd	6.6	RGV	Home	-	<20	-	-	-	<20	-	-	2012-1:80	-	-	-	-
	198	F	Belgian Malinois	8.9	Laredo	Home	-	<20	+	-	-	<20	+	-		-	-	-	-
	227	M	German Shepherd	8.9	Del Rio	Home	-	<20	-	-	-	<20	-	-	2013-1:20	-	-	-	-
Negative	147	M	Dutch Shepherd	8.1	Laredo	Kennel	-	<20	-	-	-		-	-		-	-	-	-
	164	M	Lab	6.0	Laredo	Kennel	-	<20	-	-	-		-	-		-	-	-	-
	168	F	Belgian Malinois	8.2	Laredo	Home	-			-	-		-	-		-	-	-	-
	210	M	Belgian Malinois	7.1	Del Rio	Kennel	-	<20	-	-	-		-	-		-	-	-	-
	257	F	Belgian Malinois	10.0	Del Rio	Home	-			-	-		-	-		+	-	-	-
	236	F	Belgian Malinois	8.2	Del Rio	Home	-			-	-		-	-		-	-	-	-
	245	M	German Shepherd	5.1	Del Rio	Kennel	-	<20	-	-	-		-	-		-	-	-	-
	48	M	Belgian Malinois	4.4	RGV	Kennel	-			-	-		+	-		-	-	-	-
	58	F	Belgian Malinois	7.5	RGV	Home	-			-	-		-	-		-	-	-	-
	180	F	Groenendael	4.9	Laredo	Home	-			-	-		-	-		-	-	-	-
	11	M	Dutch Shepherd	3.3	RGV	Home	+	<20	-	-	-	<20	+	-		-	-	-	-

	94	F	Belgian Malinois	7.5	RGV	Home	-			-	-		-	-		-	-	-	-
	59	F	Belgian Malinois	7.0	RGV	Home	-	<20	-	-	-		-	-		-	-	-	-
	530	M	Belgian Malinois	5.2	Del Rio	Home	N/A	N/A	N/A	N/A	-	<20	+	-		-	-	-	-
	16	M	German Shepherd	7.6	RGV	Home	-	<20	-	-	-		-	-		-	-	-	-
	193	F	German Shepherd	5.2	Laredo	Home	-			-	-		+	-		-	-	-	-
	54	M	Belgian Malinois	3.9	RGV	Kennel	-			-	-		+	-		-	-	-	-
	258	M	German Shepherd	4.0	Del Rio	Home	-	<20	-	-	-		-	-		-	-	-	-
Positive	238	F	Belgian Malinois	7.9	Del Rio	Home	+	<20	+	-	-	<20	+	-		-	-	-	-
	369	F	Belgian Malinois	7.2	RGV	Home	+	<20	+	-	+	<20	+	-		-	-	-	-
	61	M	Belgian Malinois	3.5	RGV	Home	+	<20	+	-	+	<20	+	-		-	-	-	-
	10	M	Belgian Malinois	3.9	RGV	Home	+	<20	+	-	+	<20	+	-		-	-	-	-
	252	M	Dutch Shepherd	5.3	Del Rio	Home	+	160	+	-	+	320	+	-		-	-	-	-
	200	M	Sable Shepherd	8.3	Del Rio	Home	+	20	+	-	-	<20	-	-		-	-	-	-
	66	M	Belgian Malinois	7.3	RGV	Home	-	<20	+	-	-	<20	+	-	2012-1:20	+	-	-	-
	159	F	German Shepherd	4.8	Laredo	Kennel	+	640	+	-	+	320	+	-		-	-	-	-
	62	M	German Shepherd	6.1	RGV	Home	n/a	n/a	n/a	n/a	+	640	+	-	2013-1:20	-	-	-	-
	53	M	Belgian Malinois	5.8	RGV	Home	-	<20	+	-	+	<20	-	-	2013-1:20	-	-	+	-
	203	M	Sable Shepherd	5.4	Del Rio	Home	+	320	+	-	+	320	+	-		-	-	-	-
	105	M	German Shepherd	7.4	Laredo	Home	+	320	+	-	+	320	+	-		-	-	-	-
	60	M	German Shepherd	7.1	Del Rio	Home	n/a	n/a	n/a	n/a	+	320	+	-	2016-1:8192	-	-	-	-

	153	M	Lab	5.1	Laredo	Kennel	**+**	**<20**	**+**	**-**	**-**	**<20**	**+**	**-**		**-**	**-**	**-**	**-**
	65	M	German Shepherd	8.0	RGV	Home	**+**	**320**	**+**	**-**	**+**	**320**	**+**	**-**		**-**	**-**	**-**	**-**
	92	F	Belgian Malinois	8.4	RGV	Home	**+**	**320**	**+**	**-**	**+**	**320**	**+**	**31.32 (Tcl)**		**-**	**-**	**-**	**-**
	64	F	Belgian Malinois	7.8	RGV	Home	**+**	**<20**	**+**	**-**	**+**	**<20**	**+**	**-**		**-**	**-**	**-**	**-**
